# Does intensity modulation increase target dose calculation errors of conventional algorithms for lung SBRT?

**DOI:** 10.1002/acm2.12266

**Published:** 2018-02-01

**Authors:** Dandan Zheng, Vivek Verma, Shuo Wang, Xiaoying Liang, Sumin Zhou

**Affiliations:** ^1^ Department of Radiation Oncology University of Nebraska Medical Center Omaha NE USA; ^2^ University of Florida Health Proton Therapy Institute Jacksonville FL USA

**Keywords:** IMRT, lung, Monte Carlo, SBRT, VMAT

## Abstract

**Purpose:**

Conventional dose algorithms (Type A and Type B) for lung SBRT can display considerable target dose errors compared to Type‐C algorithms. Intensity‐modulated techniques (IMRT/VMAT) are increasingly being utilized for lung SBRT. Therefore, our study aimed to assess whether intensity modulation increased target dose calculation errors by conventional algorithms over conformal techniques.

**Methods:**

Twenty lung SBRT patients were parallely planned with both IMRT and dynamic conformal arc (DCA) techniques using a Type‐A algorithm, and another 20 patients were parallely planned with IMRT, VMAT, and DCA using a Type‐B algorithm. All 100 plans were recalculated with Type‐C algorithms using identical beam and monitor unit settings, with the Type‐A/Type‐B algorithm dose errors defined using Type‐C recalculation as the ground truth. Target dose errors for PTV and GTV were calculated for a variety of dosimetric end points. Using Wilcoxon signed‐rank tests (*p* < 0.05 for statistical significance), target dose errors were compared between corresponding IMRT/VMAT and DCA plans for the two conventional algorithms. The levels of intensity modulation were also evaluated using the ratios of MUs in the IMRT/VMAT plans to those in the corresponding DCA plans. Linear regression was used to study the correlation between intensity modulation and relative dose error magnitudes.

**Results:**

Overall, larger errors were found for the Type‐A algorithm than for the Type‐B algorithm. However, the IMRT/VMAT plans were not found to have statistically larger dose errors from their corresponding DCA plans. Linear regression did not identify a significant correlation between the intensity modulation level and the relative dose error.

**Conclusion:**

Intensity modulation did not appear to increase target dose calculation errors for lung SBRT plans calculated with conventional algorithms.

## 1 INTRODUCTION

Stereotactic body radiotherapy (SBRT) is an increasingly common treatment for patients with inoperable early stage non‐small cell lung cancer (NSCLC) and other lung tumors, with well‐demonstrated efficacy and minimal side effects.^1^, ^2^, ^3^ Accurate dose calculation during treatment planning is especially important in SBRT, owing to high‐fractional doses delivered over a small number of fractions. However, two factors unique to lung SBRT increase the difficulty of achieving this necessary accuracy. First, tissue heterogeneity between the high‐density tumor and the surrounding low‐density lung tissue complicates dose calculations owing to the loss of charged particle equilibrium.^4^ Second, the small field sizes associated with the small target volume further exacerbate this problem.^5^, ^6^, ^7^

The obstacle of dose calculation has been addressed by the development of more accurate dose calculation algorithms in commercial treatment planning systems, from earlier homogeneous dose calculations to newer dose algorithms incorporating heterogeneity correction.^4^ These latter algorithms are usually categorized from Type A to Type C, with increasing dose calculation accuracies:^8^, ^9^ (1) Type A: algorithms with a one‐dimensional equivalent path length correction such as pencil beam (PB) convolution and ray tracing;^5^, ^7^, ^10^ (2) Type B: algorithms applying two‐dimensional corrections such as collapsed cone convolution (CCC)^11^ and analytical anisotropic algorithm (AAA);^12^ and (3) Type C: advanced algorithms such as fast Monte Carlo algorithms^4^, ^13^ and Boltzmann Solver‐based algorithms such as acuros external beam (AXB).^9^

Dose calculation has been widely compared among the above algorithms for lung SBRT, with interalgorithm differences mostly observed for the target dose.^5^, ^7^, ^9^, ^10^, ^14^ It is known that as compared with Type‐C algorithms, Type‐A and Type‐B algorithms tend to overestimate the target dose for lung SBRT, and the magnitude of the dose error varies widely from case to case, up to over 30% for Type A and over 15% for Type B.^7^, ^9^, ^15^ These results include both forward plans using conventional conformal techniques as well as inverse plans using modern intensity‐modulated techniques. Early lung SBRT primarily employed conformal techniques such as the 3D conformal beam technique and the dynamic conformal arc technique.^1^, ^16^ However, in recent years, intensity‐modulated techniques such as intensity‐modulated radiation therapy (IMRT) and volumetric‐modulated arc therapy (VMAT) have gained increasing popularity owing to superior organ‐at‐risk sparing, dose conformity, and fast delivery in the case of VMAT.^9^, ^17^

Also contributing to these paradigm shifts where recent studies displaying that the dominant effect of the “interplay” between tumor/tissue motion and multileaf collimator (MLC) modulation involved the blurring of dose distributions, which appeared to be small.^18^, ^19^ However, other concerns remain regarding the suitability of intensity‐modulated techniques for lung SBRT treatments, associated with smaller field apertures, and specifically whether they would lead to significantly higher dose errors than conformal techniques when calculated with conventional dose algorithms. This concern was based on previous findings that field size impacts dose errors (wherein smaller target sizes were associated with higher dose errors than larger target sizes).^5^, ^7^, ^14^, ^20^ However, this intuitive concern has not been substantiated; the dose error influence of intensity modulation, which effectively generates smaller field apertures, remains to be elucidated.

In this study, we designed a back‐to‐back comparison to study the impact of intensity modulation on dose errors of conventional Type‐A and Type‐B algorithms. The target dose errors of a Type‐A algorithm were statistically compared between IMRT and conformal techniques, and those of a Type‐B algorithm were compared between VMAT, IMRT, and conformal techniques.

## 2 MATERIALS AND METHODS

### 2.A Patient simulation and contouring

Under the approval of the University of Nebraska Medical Center Institutional Review Board, 40 patients with early stage non‐small cell lung cancer (NSCLC) treated with lung SBRT at our institution between June 2012 and August 2016 were randomly selected for this retrospective study. The simulation and contouring process was as previously described.^15^ Briefly, patients were simulated with a free‐breathing 3D CT followed by a 4D CT. The gross tumor volume (GTV) was delineated using only the 3D CT, while the internal target volume (ITV) was delineated using both the 4D and 3D CTs, and the planning target volume (PTV) was generated by adding an isotropic expansion of 5 mm to the ITV.

### 2.B Patient grouping and treatment planning

Of the 40 randomly selected patients, 20 were used for the Type‐A algorithm study and the other 20 were used for the Type‐B algorithm study. A TrueBeam STx linear accelerator equipped with an HD MLC (Varian Medical Systems, Palo Alto, CA, USA), which has a 2.5 mm leaf width for centrally located MLCs, was used to plan all patients. Beam energies included 6 MV, 6 MV flattening filter free (FFF), and 10 MV FFF for different patients. For different parallel plans of the same patient, identical beam energies were always used. All plans were generated using conventional dose algorithms according to the dosimetric constraints of the RTOG 0813 and RTOG 0915 protocols^21^, ^22^ and normalized to 95% of PTV receiving 100% of the prescription dose, which was either 48 Gy in four fractions or 50 Gy in five fractions.

For the Type‐A study, a dynamic conformal arc (DCA) plan as previously described and an IMRT plan were created for each patient using a pencil beam algorithm with equivalent path length heterogeneity correction in iPlan v4.5 (Brainlab AG, Feldkirchen, Germany).^15^ Both DCA and IMRT plans used a 360° geometry, with a full arc for DCA and seven equal angularly spaced beams for IMRT. Dose errors were compared between the two plan modalities for the Type‐A algorithm, using recalculated dose (with identical beam and monitor unit settings) by the Voxel Monte Carlo (VMC) algorithm in iPlan v4.5 as the ground truth. For VMC calculation, the full MLC geometry simulation “Accuracy Optimized Model,” with a spatial resolution of 2 mm and variance of 1%, was used.

For the Type‐B study, a DCA plan, a VMAT plan as previously described,^9^ and an IMRT plan utilizing the same planning objectives were created for each patient using the AAA algorithm in Eclipse v13.5 (Varian Medical Systems, Palo Alto, CA, USA). Most patients had full‐arc DCA and VMAT plans, together with seven‐beam IMRT plans equally spaced over 360°. Two patients with very peripheral lesions had partial‐arc DCA and VMAT plans, together with seven‐beam IMRT plans equally spaced over the same partial arc. Dose errors were compared between the three plan modalities for the Type‐B algorithm, using recalculated dose (with identical beam and monitor unit settings) by the AXB algorithm in Eclipse as the ground truth.

A dose grid of 2 mm was used for all of the above calculations. Dose to medium was used for both VMC and AXB calculations.

### 2.C Dosimetric data acquisition and analysis

For each of the above described plans on each patient, target dose end points were recorded including the minimum (Dmin), mean (Dmean), and maximum (Dmax) dose of the PTV and the GTV as well as the dose to 5% (D5%) and 95% (D95%) of the PTV and GTV volumes. A MATLAB script (MathWorks Inc., Natick, MA, USA) was utilized to assist parameter extraction from the exported dose–volume histogram data. For each plan, the relative dose errors were defined as the percentage difference of the dose end point calculated by the conventional algorithm (Type A or Type B) relative to the corresponding Type‐C recalculation. The relative errors were then compared between IMRT and DCA for the Type‐A algorithm and between both VMAT and DCA as well as IMRT and DCA for the Type‐B algorithm. Statistical analysis for each comparison used the Wilcoxon signed‐rank test on R Studio software. A *p* < 0.05 was considered statistically significant.

### 2.D Intensity modulation vs relative dose errors

For each IMRT or VMAT plan, the level of intensity modulation was evaluated using the modulation ratio defined as the ratio of the total monitor units to those of the corresponding DCA plan. For all 60 intensity‐modulated plans (20 Type‐A IMRT plans, 20 Type‐B IMRT plans, and 20 Type‐B VMAT plans), the relative dose errors, defined as the differences between dose errors of the intensity‐modulated plans and those of the corresponding DCA plans, were calculated. Linear regression was used to evaluate whether the relative dose errors were dependent on the levels of intensity modulation.

## 3 RESULTS

### 3.A Patient, tumor, and plan characteristics

Patient, tumor, and plan characteristics of the 40 patients are described in Table 1. A median modulation ratio of 1.4, 1.4, and 1.5 was calculated for Type‐A IMRT plans, Type‐B VMAT plans, and Type‐B IMRT plans, respectively.

**Table 1:** Patient, tumor, and plan characteristics

Parameter	Total
Patients (*n* = 40)	21 female, 19 male
Median age, years (range)	73 (55–95)
Median PTV, cm^3^ (range)	36.2 (8.8–121.3)
Tumor location (*n* = 40)	10 LUL, 10 RUL, 12 LLL, 7 RLL, 1 RML
Type‐A IMRT plan median modulation ratio (range)	1.5 (1.2–1.9)
Type‐B VMAT plan median modulation ratio (range)	1.6 (1.2–2.0)
Type‐B IMRT plan median modulation ratio (range)	1.6 (1.2–2.2)

PTV, planning target volume; LUL, left upper lobe; RUL, right upper lobe; LLL, left lower lobe; RLL, right lower lobe; RML, right middle lobe; IMRT, intensity‐modulated radiation therapy; VMAT, volumetric‐modulated arc therapy.

### 3.B Type‐A dose errors

As listed in Table 2, the Type‐A calculation led to a target dose overestimation for both the PTV and GTV for all dose end points on all plans. The dose error magnitude varied widely from case to case, from a few percentage to over 30%. In general, the dose errors were larger for target “cold spot” metrics such as Dmin and D95% than for target “hot spot” parameters such as Dmax and D5%. They were also larger for the PTV than for the GTV. Comparing between IMRT and DCA plans, most dose end points displayed no statistically different dose errors. For a couple end points with p<0.05, the absolute dose differences (where IMRT dose errors were numerically smaller than those for DCA) were very small and clinically insignificant.

**Table 2:** Median (range) of percentage PTV and GTV dose errors in Type‐A dose calculations comparing IMRT and DCA for the 20 patients. The bolded *p* values indicate statistically significant differences for the high‐dose region endpoints D5% and Dmax of both PTV and GTV, where IMRT resulted in significant lower dose errors than DCA (*p* < 0.05)

	PTV Dmin	PTV D95%	PTV Dmean	PTV D5%	PTV Dmax
IMRT	17.6% (13.2–36.9%)	15.2% (12.1–32.7%)	9.1% (6.4–17.1%)	4.3% (1.2–8.9%)	2.9% (1.1–8.2%)
DCA	23.1% (14.5–34.2%)	16.1% (14.4–33.1%)	7.6% (6.2–17.9%)	5.1% (1.9–10.1%)	4.1% (1.8–8.5%)
*p* value	0.46	0.62	0.57	**0.0002**	**0.003**

	GTV Dmin	GTV D95	GTV D50	GTV D5	GTV Dmax
IMRT	12.2% (6.4–24.7%)	6.9% (3.8–14.8%)	6.1% (2.9–9.3%)	3.3% (2.1–6.9%)	2.9% (1.1–8.1%)
DCA	9.8% (2.3–16.8%)	7.0% (4.1–13.2%)	5.3% (3.1–8.7%)	4.0% (3.0–7.2%)	3.7% (1.2–8.5%)
*p* value	0.45	0.86	0.43	**0.0013**	**0.008**

### 3.C Type‐B dose errors

Compared with Type‐A dose errors, Type‐B dose algorithm errors were overall smaller in magnitude. For example, the average PTV D95% error of the Type‐A calculation was 7.5%, whereas that of the Type‐B calculation was 1.7%. Similar to the wide magnitude of variation among cases observed in Type‐A dose errors, Type‐B dose errors also varied widely in range. Unlike the Type‐A algorithm which overestimated all dose end points for all patients, the Type‐B algorithm in general overestimated the target “cold spot” indices and underestimated the target “hot spot” parameters. For the target “cold spot” metrics such as Dmin and D95%, the Type‐B calculation in most cases overestimated the dose for up to 17.9% for Dmin and 10.6% for D95%, but it also sometimes led to slight underestimations within 3% for these dose endpoints in other cases. Detailed dose error results are listed in Table 3.

**Table 3:** Median (range) of percentage PTV and GTV dose errors in Type‐B dose calculations comparing IMRT vs DCA and VMAT vs DCA for the 20 patients

	PTV Dmin	PTV D95%	PTV Dmean	PTV D5%	PTV Dmax
IMRT	3.8% (−0.8 to 17.9%)	2.4% (−1.9 to 11.3%)	1.1% (−2.8 to 5.1%)	−2.3% (−5.8 to 0.9%)	−2.7% (−5.2 to 1.0%)
VMAT	3.5% (−0.6 to 13.3%)	2.1% (−0.6 to 10.4%)	1.2% (−3.0 to 4.8%)	−1.8% (−5.5 to 1.1%)	−2.2% (−5.8 to 1.3%)
DCA	3.1% (−1.2 to 10.8%)	1.2% (−2.9 to 9.1%)	0.5% (−1.8 to 4.2%)	−0.4% (−4.2 to 0.9%)	−0.7% (−4.4 to 1.6%)
*p* value (IMRT vs DCA)	0.82	0.51	0.67	0.84	0.74
*p* value (VMAT vs DCA)	0.56	0.39	0.78	0.65	0.43

	GTV Dmin	GTV D95	GTV D50	GTV D5	GTV Dmax
IMRT	1.6% (−0.5 to 10.3%)	1.2% (−0.3 to 6.4%)	0.1% (−1.3 to 3.2%)	−1.2% (−3.5 to 0.8%)	−1.8% (−4.7 to 0.8%)
VMAT	1.9% (−0.3 to 8.9%)	0.9% (−0.5 to 6.7%)	0.3% (−1.8 to 3.5%)	−0.9% (−3.2 to 0.8%)	−1.6% (−3.9 to 0.9%)
DCA	1.2% (−0.5 to 6.8%)	1.0% (−0.4 to 5.6%)	0.5% (−1.0 to 3.3%)	−0.5% (−2.5 to 0.8%)	−1.0% (−3.1 to 1.0%)
*p* value (IMRT vs DCA)	0.78	0.81	0.88	0.59	0.62
*p* value (VMAT vs DCA)	0.29	0.87	0.67	0.75	0.71

Comparing between IMRT and DCA or between VMAT and DCA, the “cold spot” parameters such as Dmin and D95% had, on average, larger numeric (but statistically insignificant) errors for IMRT or VMAT than for their DCA counterparts.

### 3.D Intensity modulation vs relative dose errors

**Figure 1:**
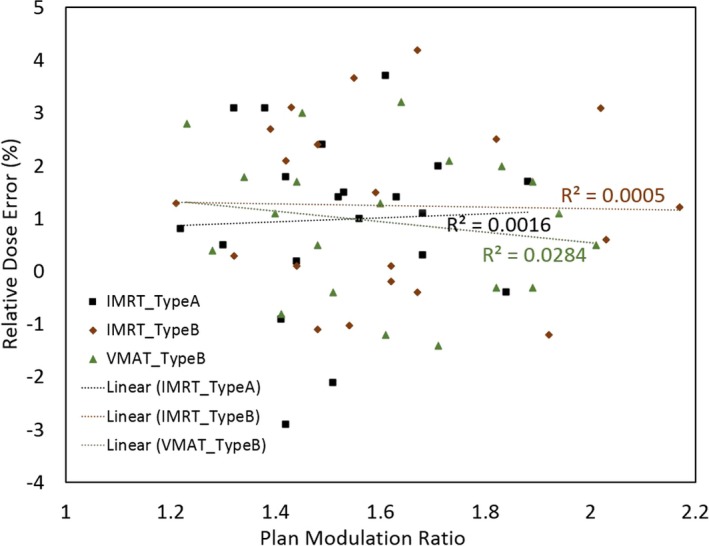
The relative dose error of PTV D95% and the modulation ratio of the plan for the 60 intensity‐modulated plans. Linear regression results are also shown for each plan type.

As listed in Table 1, the levels of intensity modulation were moderate for the 60 intensity‐modulated plans investigated in the study, with modulation ratios between 1.2 and 2.2. The relative dose errors did not appear to correlate with the levels of plan intensity modulation. For example, a scatter plot is depicted in Fig. 1 to illustrate the relationships between the relative dose error of PTV D95% and the modulation ratio of the plan for the 60 intensity‐modulated plans. As indicated by the low *R* ^2^ values of the linear regression, a clear dependence between the relative dose errors and the plan modulation ratios was not observed.

## 4 DISCUSSION

In this work, we investigated the impact of intensity modulation on target dose errors of conventional Type‐A and Type‐B dose algorithms relative to the more accurate Type‐C algorithms on 20 randomly selected lung SBRT patients for each algorithm. For the Type‐A algorithm, parallel‐planned IMRT and DCA plans were compared. For the Type‐B algorithm, both IMRT plans and VMAT plans were created to compare against DCA plans. While the dose errors of Type‐A and Type‐B algorithms have been reported in previous studies for lung SBRT plans using 3D conformal techniques such as DCA or intensity‐modulated techniques such as IMRT and VMAT,^5^, ^7^, ^9^, ^14^, ^15^ this is, to our knowledge, the first study to design a parallel comparison to investigate whether intensity modulation would increase target dose errors for these conventional‐dose algorithms. This investigation is important because intensity‐modulated techniques are increasingly used for lung SBRT planning owing to their superior organ‐at‐risk sparing, improved efficiency in the case of VMAT, together with the eased concerns about the motion interplay effect in light of recent findings.^18^ At the same time, the dose calculation accuracy issue has also been increasingly recognized for lung SBRT. To exemplify this, NRC‐RTOG protocols have changed from using homogeneous calculation to recommending heterogeneity correction with sophisticated dose algorithms for this treatment;^21^, ^22^ and numerous studies have been, or are being, conducted to study dose errors of conventional algorithms as well as to implement new algorithms. Type‐B algorithms are the current staple in terms of clinical treatment planning algorithms in use, but Type‐A algorithms still play a significant role for clinical lung SBRT treatment planning.^23^ Therefore, this investigation on whether intensity‐modulated techniques make these algorithms more susceptible to target dose errors is timely and important.

Intuitively, intensity modulation is expected to increase the target dose errors of conventional algorithms. This is because the loss of charged particle equilibrium is more severe for smaller field sizes, making it more challenging for conventional dose algorithms to accurately calculate the dose. Indeed, several previous studies have found that smaller target volumes — hence smaller field sizes — tend to result in larger target dose errors.^5^, ^7^, ^20^ Intensity modulation effectively decreases the field size for a given target, through dose painting with small beamlets. However, our study did not find significant increases of target dose errors with intensity modulation for either Type‐A or Type‐B algorithms. This may seem counterintuitive at first glance, but we stipulate that it may result from the small beamlets in IMRT usually being at the center of the target instead of at the periphery. It is at the target periphery that conventional algorithms have more inaccurate dose modeling, owing to the inhomogeneous interfaces between the low‐density lung tissue and the high‐density tumor tissue. Therefore, although smaller effective fields or beam apertures are used in intensity‐modulated plans, they are not at the target periphery, thereby not significantly increasing the calculated target dose errors. Our study also did not find any linear correlation between the plan modulation ratio and the relative dose error of the modulated plan over the conformal plan. There is likely a complex relationship between the specific beamlets in a modulated plan in terms of location and size and their dose error contributions due to heterogeneity. It is therefore speculated that a compounded and nonlinear relationship existed between the total relative dose error and the overall modulation ratio.

The dose errors observed in this study for Type‐A and Type‐B algorithms are in agreement with numerous previous studies.^7^, ^9^, ^14^, ^15^ Both algorithms overestimated the target peripheral or “cold spot” indices such as D95% and Dmin, and the magnitude of the overestimation varied widely from case to case. The Type‐A algorithm errors were in general larger up to over 30% for PTV D95%, making the algorithm unsuitable for lung SBRT planning. The Type‐B algorithm errors were smaller for most cases, but could be up to 11% for some cases, which may necessitate the use of more accurate Type‐C algorithms going forward.

In our study, the VMAT technique was only investigated for the Type‐B algorithm (not for the Type‐A algorithm) because the treatment planning system iPlan utilized for the Type‐A investigation does not support VMAT planning. Another limitation of our study was that only one Type‐A and one Type‐B algorithm were investigated; therefore, the results and trends found may or may not be generalizable to other algorithms. Herein, the Type‐B algorithm utilized was AAA, which may be less accurate than another Type‐B algorithm, CCC.^24^, ^25^, ^26^ However, it should also be noted that AAA is still quite prevalent in the current clinical treatment planning, which makes this study quite relatable to contemporary practice. Finally, our study used DCA as the conformal technique for comparison. While DCA is a popular conformal technique used for lung SBRT,^9^, ^10^, ^14^, ^15^, ^16^ other techniques are also used such as multiple noncoplanar conformal beams.

## 5 CONCLUSIONS

The impact of intensity modulation on conventional algorithm dose errors for lung SBRT was investigated herein. Intensity modulation does not appear to increase the target dose errors for lung SBRT plans calculated with conventional algorithms.

## CONFLICTS OF INTEREST

The authors have no relevant conflicts of interest to disclose.
